# Supplementary material for: The Antioxidant Drug Edaravone Binds to the Aryl Hydrocarbon Receptor (AHR) and Promotes the Downstream Signaling Pathway Activation
Source: Biomolecules. 2024 Apr 4;14(4):443. doi: 10.3390/biom14040443 (PMC11047889; doi:10.3390/biom14040443)
Supplement: Supplementary file 1 [file biomolecules-14-00443-s001.zip › biomolecules-2853571-Table S1.pdf]

Table S1. List of Taqman inventoried assays used for gene expression experiments

Species	Gene symbol	Assay code
Mouse	Gapdh	Mm99999915_g1
	Ahrr	Mm00477443_m1
	Cyp1a1	Mm00487218_m1
	Cyp1b1	Mm00487229_m1
Human	Gapdh	Hs99999905_m1
	Ahrr	Hs01005075_m1
	Cyp1a1	Hs01054796_g1
Zebrafish	Gapdh	Dr03436842_m1
	Cyp1a	Dr03112444_m1
	Ahrra	Dr03426356_m1
	Ahrrb	Dr03091142_m1
