# Supplementary material for: The Antioxidant Drug Edaravone Binds to the Aryl Hydrocarbon Receptor (AHR) and Promotes the Downstream Signaling Pathway Activation
Source: Biomolecules. 2024 Apr 4;14(4):443. doi: 10.3390/biom14040443 (PMC11047889; doi:10.3390/biom14040443)
Supplement: Supplementary file 1 [file biomolecules-14-00443-s001.zip › Manuscript.v8_Supplementary_Figures_revised.pdf]

The antioxidant drug edaravone binds to the aryl hydrocarbon receptor (AHR) and promotes the downstream signaling pathway activation

Caterina Veroni[1*], Stefania Olla[2*], Maria Stefania Brignone[1], Chiara Siguri[2], Alessia Formato[3], Manuela Marra[4], Rosa Manzoli[5], Maria Carla Macario[5,6], Elena Ambrosini[1], Enrico Moro[5§] and Cristina Agresti[1, §]

(1) Department of Neuroscience, Istituto Superiore di Sanità, Rome, Italy

(2) Institute for Genetic and Biomedical Research (IRGB), The National Research Council (CNR), Monserrato, Cagliari, Italy

(3) Institute of Biochemistry and Cell Biology, IBBC-CNR, Campus Adriano Buzzati Traverso, Monterotondo Scalo, Rome, Italy

(4) Core Facilities Technical-Scientific Service, Istituto Superiore di Sanità, Rome, Italy

(5) Department of Molecular Medicine, University of Padova, Padova, Italy

(6) Department of Biology, University of Padova, Padova, Italy

(*) shared first authorship

(§) corresponding author

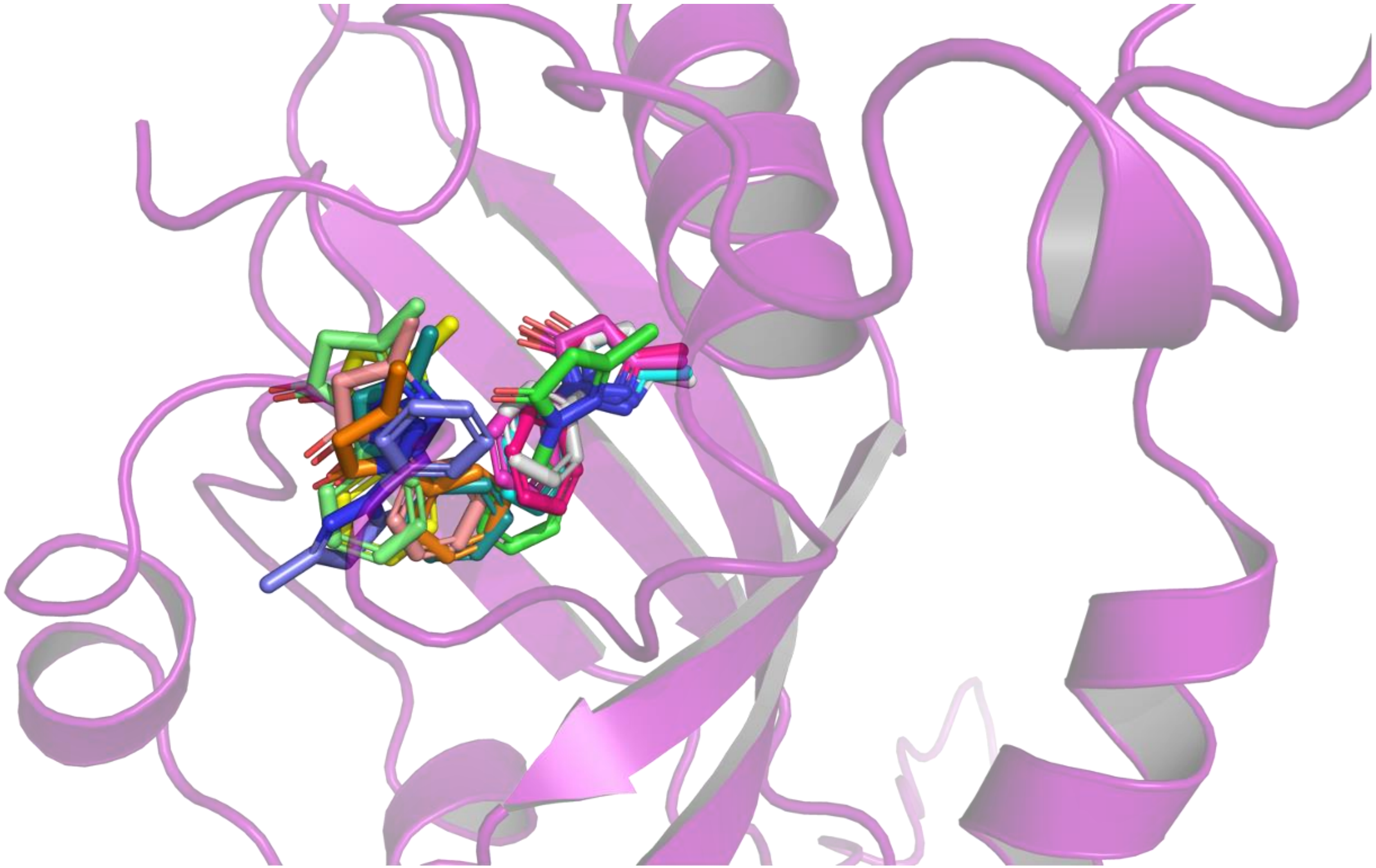

Figure S1

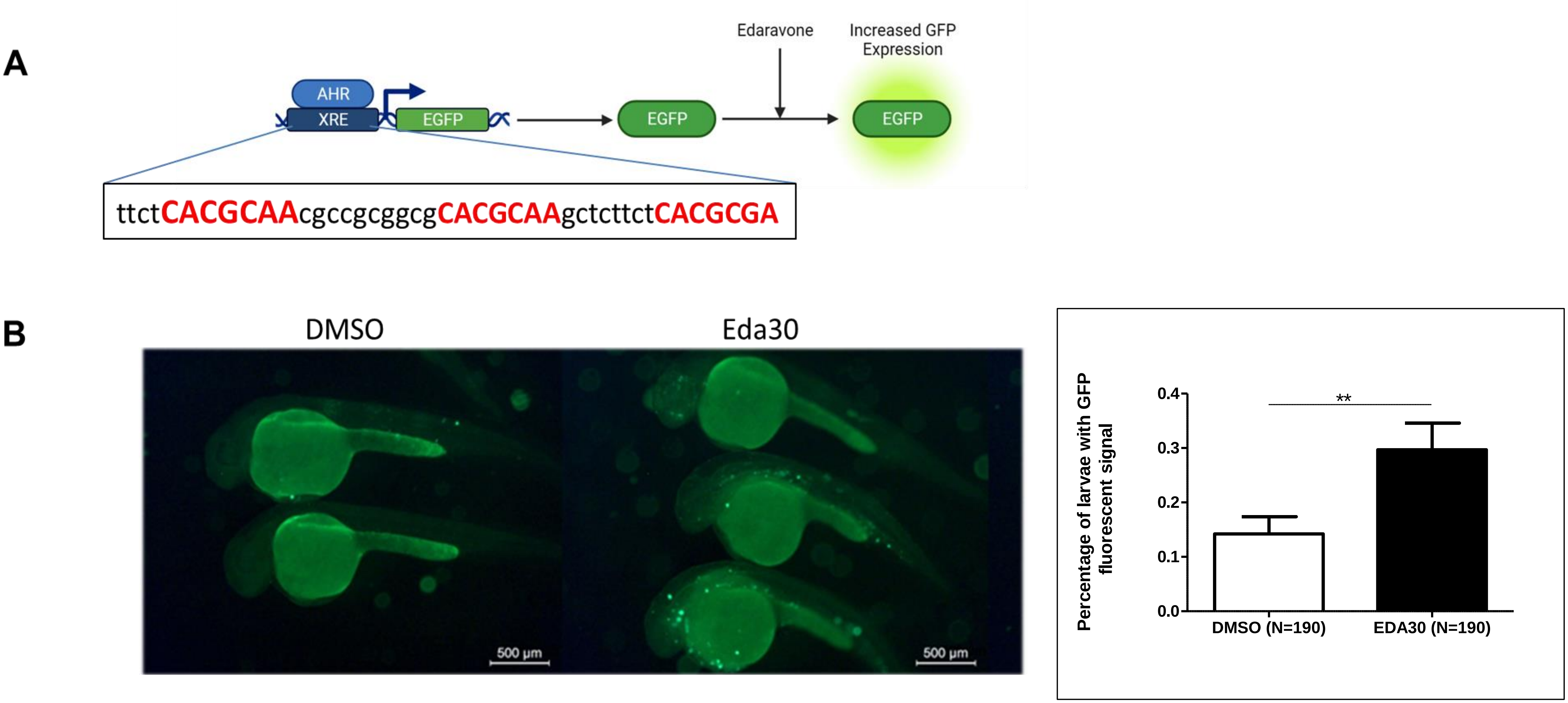
A
Edaravone
Increased GFP Expression
AHR
XRE
EGFP
EGFP
EGFP
ttctCACGCAAcgccgcggcgCACGCAAgctcttctCACGCGA
B
DMSO
Eda30
500 µm
500 µm
Percentage of larvae with GFP fluorescent signal
0.4
0.3
0.2
0.1
0.0
**
DMSO (N=190)
EDA30 (N=190)

Figure S2

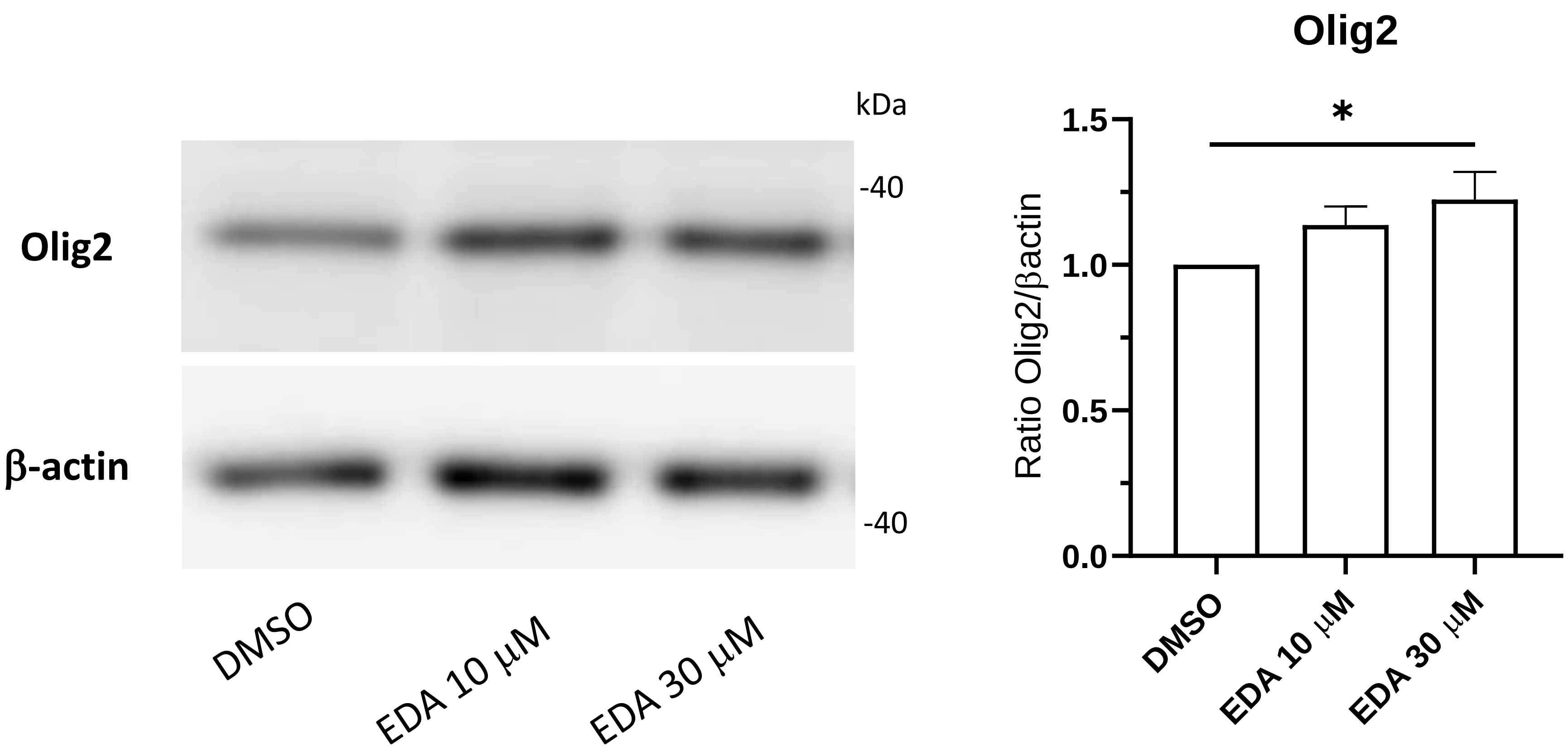
kDa
-40
Olig2
β-actin
-40
DMSO
EDA 10 μM
EDA 30 μM
Olig2
*
1.5
1.0
0.5
0.0
Ratio Olig2/βactin
DMSO
EDA 10 μM
EDA 30 μM

Figure S3

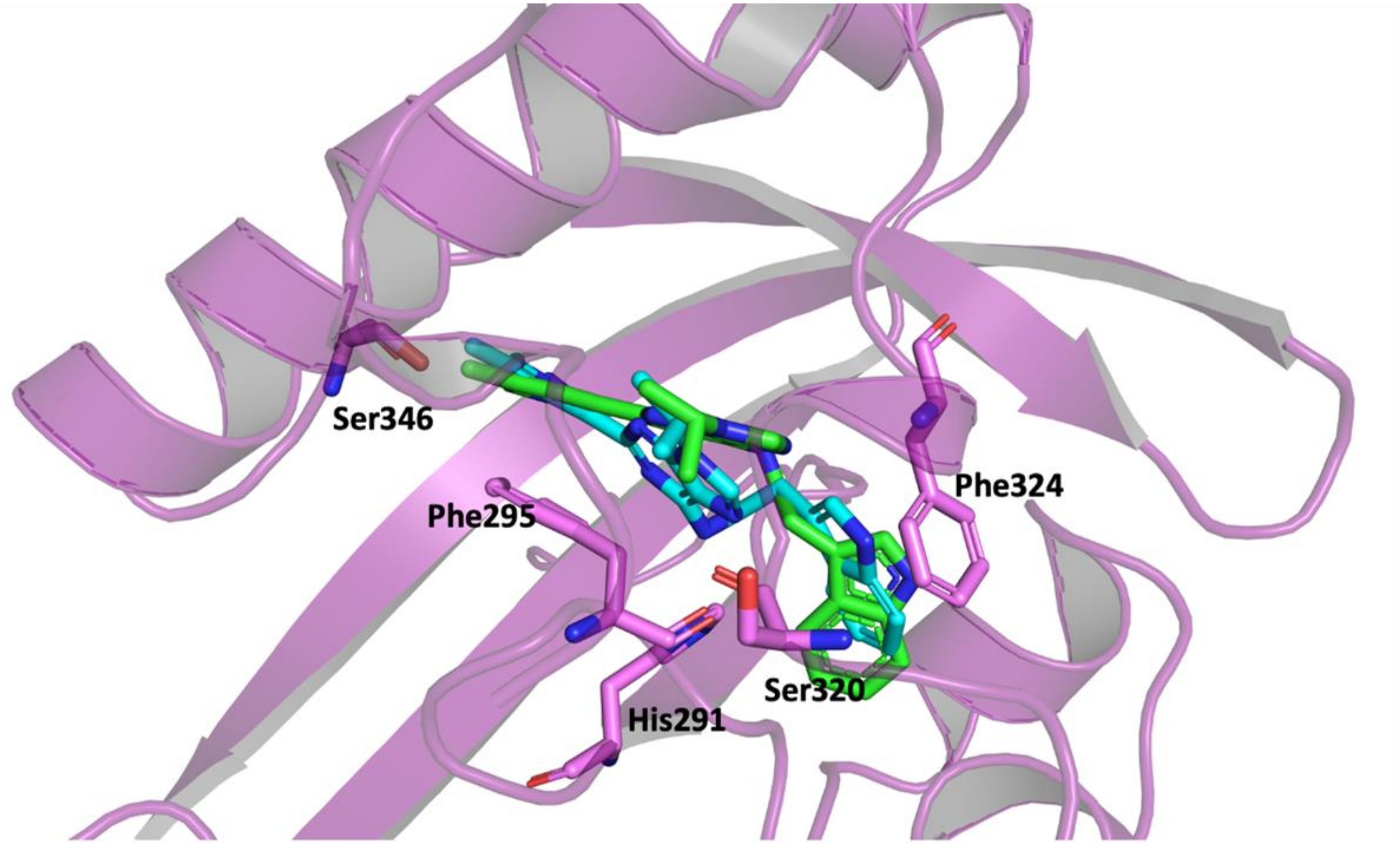

Figure S4

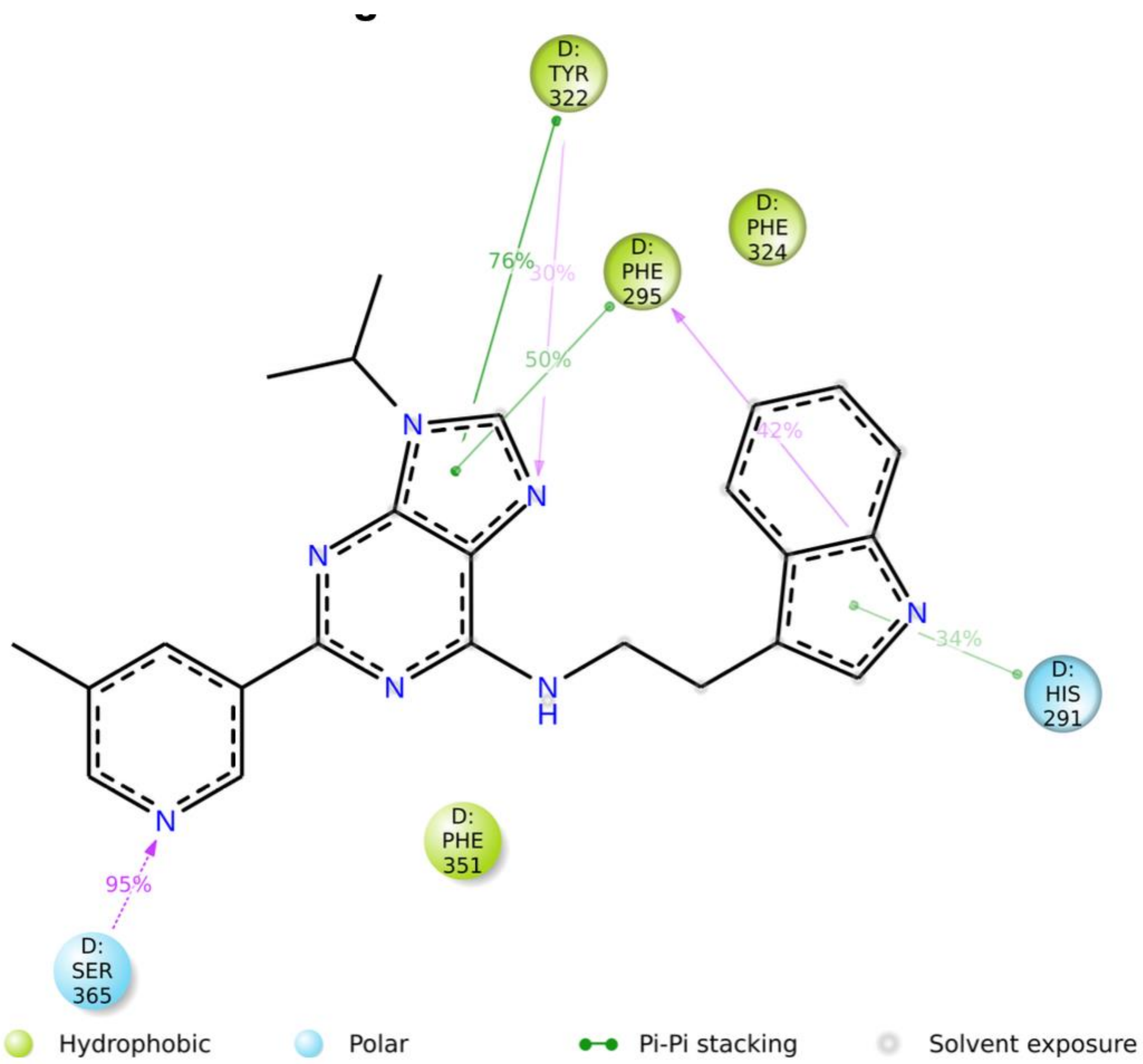
D: TYR 322
D: PHE 324
D: PHE 295
76%
30%
50%
42%
34%
D: HIS 291
D: PHE 351
95%
D: SER 365
Hydrophobic
Polar
Pi-Pi stacking
Solvent exposure

Figure S5

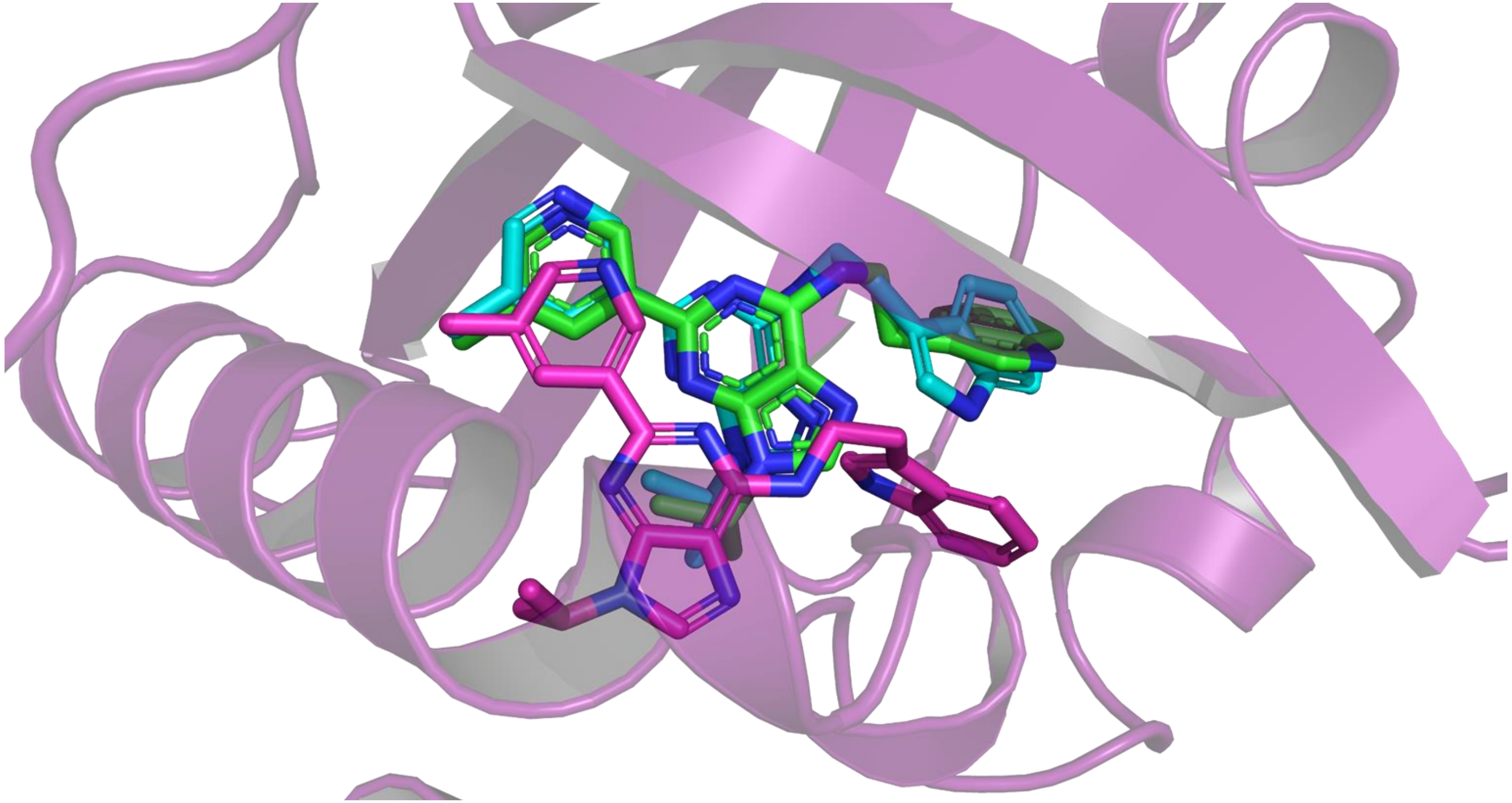

Figure S6

Figure S1. Superposition of the EDA-AHR docking pose with the clusters obtained from the molecular dynamics of EDA with AHR. Docking pose (green), cluster 1 (cyan), cluster 2 (magenta), cluster 3 (yellow), 4 (salmon), 5 (white), 6 (purple), 7 (orange), 8 (light green), 9 (forest), and 10 (hot pink).

Figure S2. EDA-induced expression of a xenobiotic responsive element (XRE)-driven reporter gene. A. Schematic picture showing the reporter cassette containing three XREs upstream of the EGFP coding sequence, which was used for *in vivo* testing. B. Representative fluorescent microscopy images, showing the transient expression of the reporter cassette in 2 dpf zebrafish larvae. Note that EDA treatment was able to induce reporter expression (fluorescent dots). All images are lateral views, with anterior to the left. **$p<0.01$ with unpaired Student's *t*-test.

Figure S3. EDA treatment increases Olig2 protein levels in fish larvae. Representative Western Blot for Olig2 in control and EDA-treated fish larvae lysates. The bar-graph depicts the quantification of detected Olig2 protein levels normalized to beta-actin. Data are expressed as the mean ±SEM of 3 biological replicates (10 larvae per replicate). *$p<0.05$ with unpaired Student's *t*-test. The image was edited using BioRender.com.

Figure S4. Overlapping poses of the docking of GNF-351 with AHR. In green the pose resulting from docking with Autodock 4, in cyan the pose resulting from docking with Glide.

Figure S5. 2D representation of the bonds above 30% that GNF-351 makes with AHR during the 500 ns of molecular dynamics.

Figure S6. Superimposing docking results and molecular dynamics of GNF-351 with AHR. In green the pose resulting from docking with Autodock 4, in cyan the pose resulting from docking with Glide, in magenta the cluster number 1 resulting from molecular dynamics simulation.
