# Supplementary material for: The Antioxidant Drug Edaravone Binds to the Aryl Hydrocarbon Receptor (AHR) and Promotes the Downstream Signaling Pathway Activation
Source: Biomolecules. 2024 Apr 4;14(4):443. doi: 10.3390/biom14040443 (PMC11047889; doi:10.3390/biom14040443)
Supplement: Supplementary file 1 [file biomolecules-14-00443-s001.zip › Manuscript.v8_Supplementary_material_Uncropped_WB.pdf]

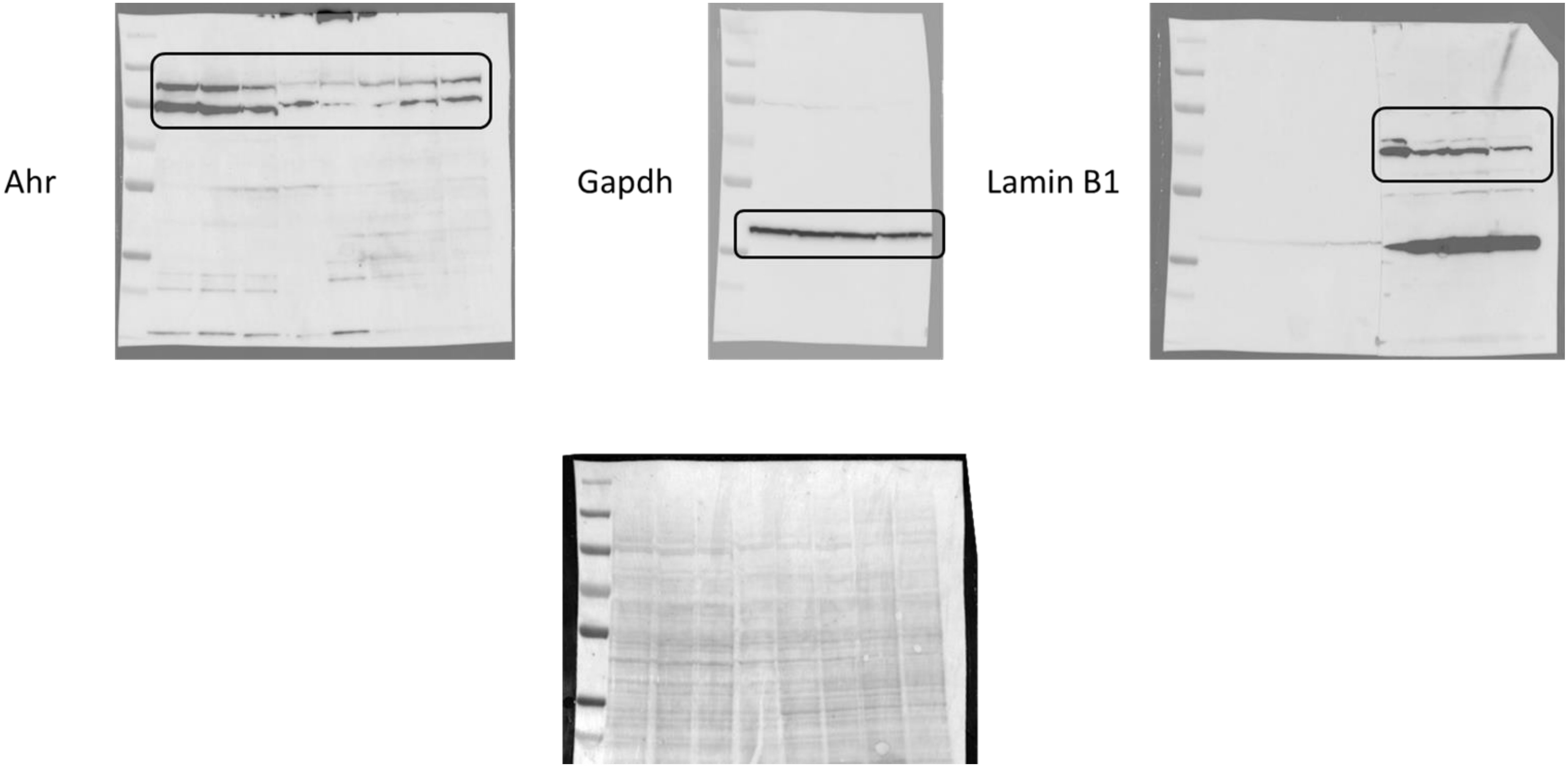

Uncropped Figure 4

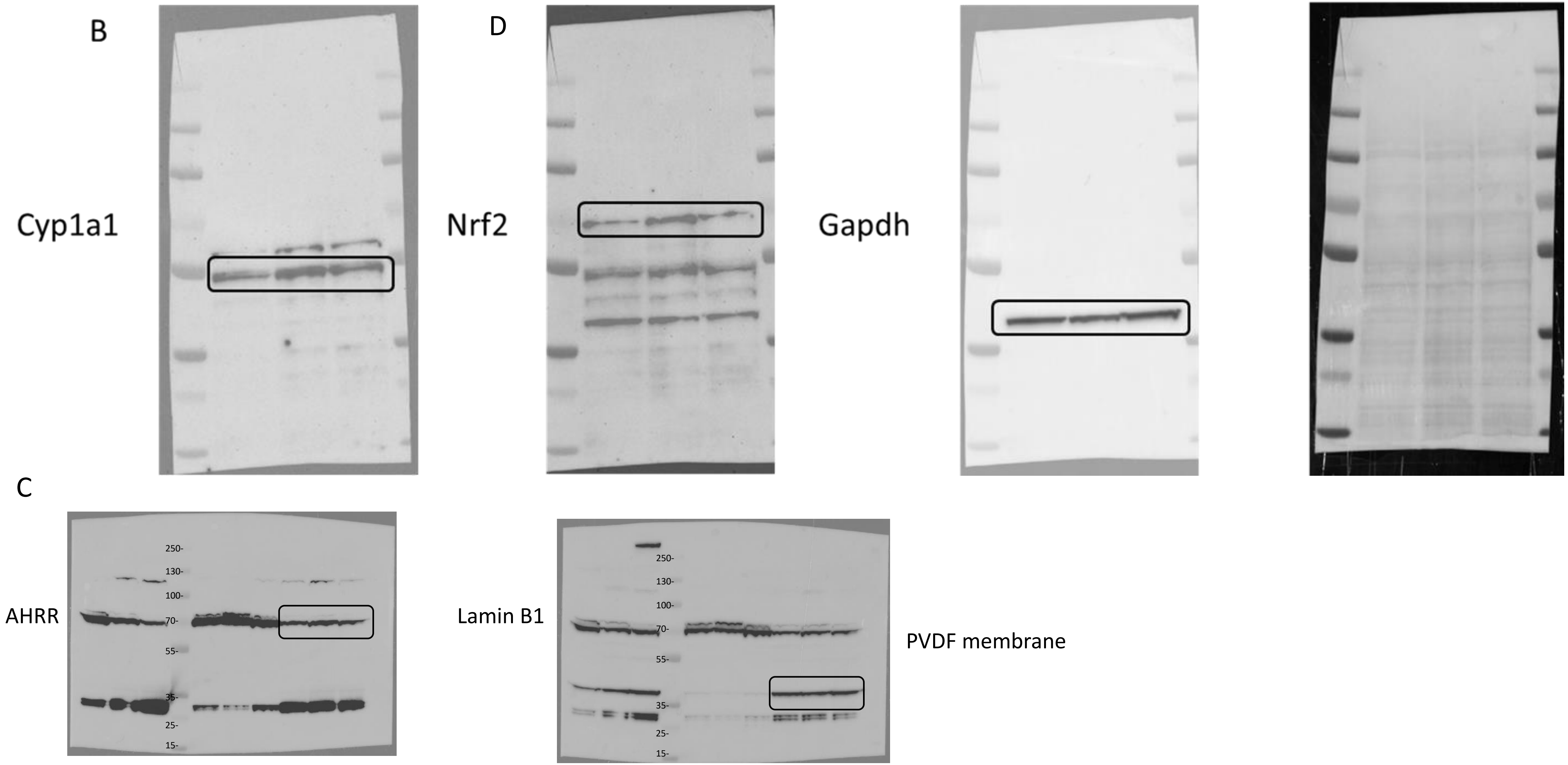

Uncropped Figure 5

GFP

β-actin

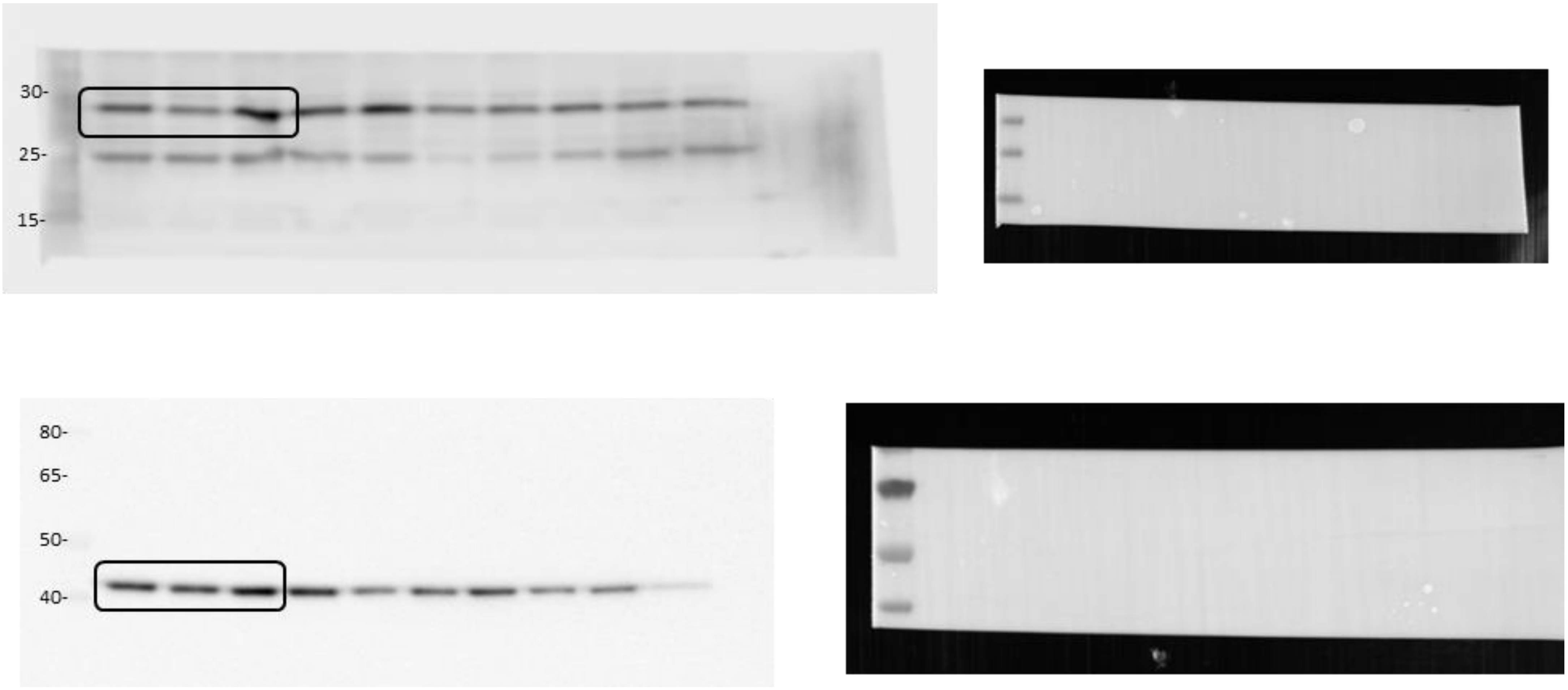

Uncropped Figure 6A

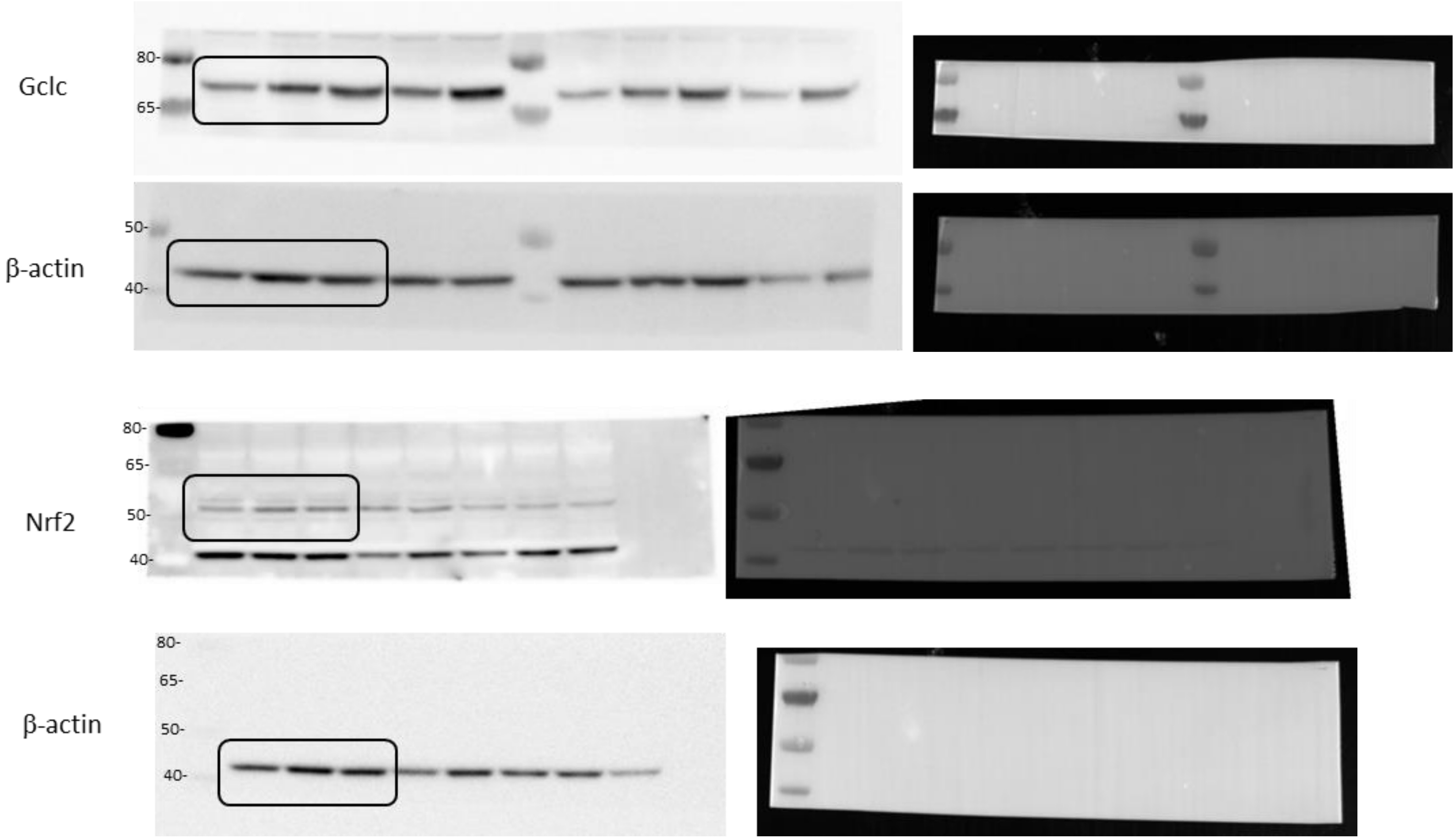

Uncropped Figure 6B and C

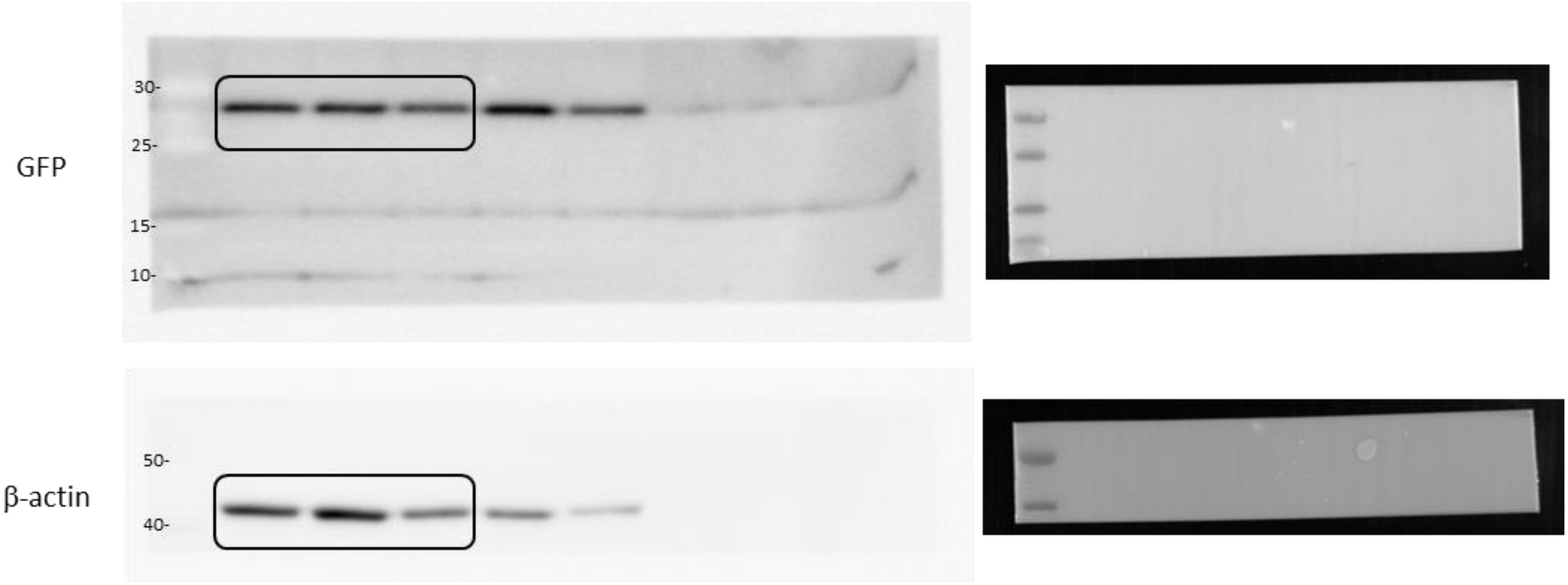

Uncropped Figure 7

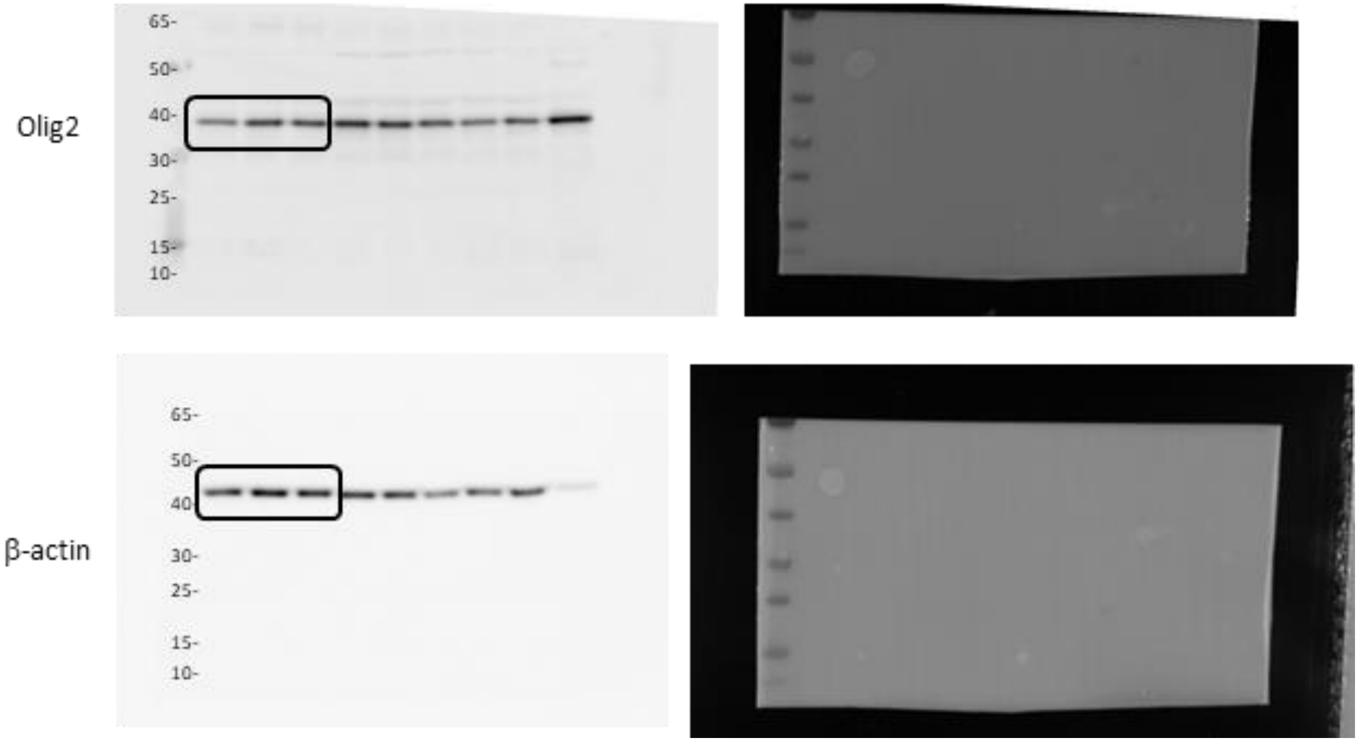

Uncropped Figure S3
